# Thyroid nodules in children - rules of management

**DOI:** 10.1186/1756-6614-8-S1-A3

**Published:** 2015-06-22

**Authors:** Irena Bobeff

**Affiliations:** 1 Department of Endocrinology and Metabolic Diseases, Polish Mother’s Memorial Research Institute, Lodz, Poland

## 

In children, thyroid lesions both palpable (nodules) and non-palpable, but visible during ultrasound (incidentaloma), require a full diagnostic procedure in accordance with the nodular goiter management algorithm. It is especially important because in children the risk of neoplastic process in thyroid nodule is 4-5 times higher than in adult patients, and reaches between 9 and 50% (on average 25%).

The pathogenesis of a goiter is not entirely known. Among the causative factors for thyroid nodules or focal lesions the literature mentions: environmental factors i.e. iodine excess or insufficiency, exposure to radiation, natural goitrogens, chemical substances (lithium carbonate), pollution (phenols, phthalates, pyridines and aromatic carbohydrates), hormonal factors (TSH, IGF-I, EGF, FGF), neuropeptides, cytokines (TGF- β), immunoglobulins (TGI) and genetic factors – TSH receptor mutation, *RAS* oncogene mutation (mainly responsible for follicular adenoma), mutations and polymorphisms of several genes including thyroid peroxidase gene, thymoglobulin gene, sodium-iodide symporter gene, *BRAF* gene, as well as mutation of genes encoding microRNA. The *DICER 1* mutation and germinal mutation of *KEAP 1* have recently been discovered.

It is also worth mentioning that during adolescence, when the requirement for thyroid hormones is increased, the thyroid gland is particularly susceptible to enlargement. Hyperglobulinemia resulting from augmented estrogen secretion in girls is another factor. It causes an increase in the pool of globulin-bound hormones and therefore relative free thyroxin (FT4) deficiency. That, in turn, leads to increased TSH levels, which is the most important factor stimulating thyroid growth.

Thyroid nodules are present in 0.2-5% of children, more frequently in girls. Morbidity increases with age. The rate of malignancy in thyroid nodules is greater in children before the age of 10 and in boys. Similarly, as in adults, papillary carcinoma is the most common thyroid malignancy.

Thyroid nodules in children demonstrate particular characteristics. Most neoplastic and non-neoplastic lesions tend to form singular nodules. Children present clinical signs such as dyspnea, dysphagia or hoarseness more rarely than adults. Patients are usually asymptomatic. Thyroid nodules in children comprise neoplastic and non-neoplastic masses, among the latter the nodular type of Hashimoto disease is the most frequent. However it is important to remember about the possibility of thyroid congenital disorders.

The main goal of thyroid nodule diagnostic process is to detect patients with malignancy risk factors. Primarily, the medical history should be obtained to gain information about the occurrence of thyroid diseases, including cancers, in patient’s family. Especially, the family history in first-degree relatives should be carefully determined for MEN (multiple endocrine neoplasm) type 1 and 2, familiar medullary thyroid cancer and familial non-medullary thyroid cancer. Medical history should also include information about exposition to therapeutic or environmental radiation, about symptoms which may indicate hyper- or hypothyroidism, the history of nodule growth (rapid growth), neck pain or any symptoms of compression. Palpation allows estimation of the size and consistency of the thyroid gland, nodules and local lymph nodes. Among complementary diagnostic methods, the main role is reserved for ultrasound-guided fine needle aspiration (FNA). Moreover, thyroid function tests should always be performed, including measuring concentrations of TSH, thyroid hormones, and anti-thyroid autoantibodies. Thyroid scintigraphy is recommended in some cases, such as preparation for radioiodine therapy of hyperthyroidism, examination of ectopic thyroid gland or diagnosis of congenital hypothyroidism. It is important to evaluate calcitonin level in the following cases: clinical suspicion of medullary cancer or confirmed *RET* mutation, non-diagnostic FNA with no further plans for surgery, FNA indicating follicular neoplasm, coexistence of thyroid nodules and adrenal gland lesions, and – finally – before each thyroid surgery, as the knowledge of calcitonin level enhances pre- and intraoperative diagnosis of medullary cancer and helps to determine the extent of the surgery.

In benign thyroid nodules that do not enlarge, it is acceptable to follow a “watch and wait” approach. Pharmacotherapy with levothyroxine (L-T4) is recommended in small, benign nodules presenting in children inhabiting iodide-deficient regions. The aim is to suppress TSH secretion. The dose of L-T4 is regulated based on TSH level, which should remain in lower limit of normal range. If the environmental iodide supply is adequate, this kind of therapy ought to be considered when anti-thyroid autoantibodies are detected. Nevertheless, thyroidectomy (subtotal or – less frequently – lobectomy) remains the most efficient therapeutic measure in thyroid nodules in children.

## Conclusions

1. In children, every thyroid nodule or incidentaloma should be evaluated according to an appropriate algorithm.

2. In each case, it is indispensable to follow thyroid function tests, as well as to assess the titer of anti-thyroid autoantibodies.

3. Ultrasound-guided FNA remains the GOLD STANDARD – the most important diagnostic method implied - in thyroid nodules both in children and adults.

4. Significant enlargement of thyroid nodule during observation or L-T4 pharmacotherapy is an urgent indication for surgery because it may indicate neoplastic growth. In such a case, FNA result implying benign lesion cannot justify abandoning surgery.

5. Thyroid goiter in children requires greater caution and more frequent control visits (at least once every 6 months) than in adults. In case of any doubt, radical treatment should be conducted.

